# Physicians’ perceptions, reported experience, and perceived structural barriers to shared decision-making in South Korea: a multispecialty survey

**DOI:** 10.3389/fpubh.2026.1938324

**Published:** 2026-10-15

**Authors:** Sang-Ho Yoo, Min Ji Kim

**Affiliations:** Department of Medical Humanities and Ethics, Hanyang University College of Medicine, Seoul, Republic of Korea

**Keywords:** health communication, health services research, implementation barriers, patient participation, patient-centered care, physicians, Republic of Korea, shared decision-making

## Abstract

**Introduction:**

Shared decision-making (SDM) is a health communication practice that supports patient participation and aligns care with patients’ values. Routine implementation depends on clinicians’ familiarity and communication practices as well as consultation time, workflow, organizational support, and financing. In this study, we examined physicians’ perceptions of patient and professional roles, reported familiarity and experience with SDM, associated physician and practice characteristics, and perceived priorities for wider implementation in South Korea.

**Methods:**

We conducted a nationally distributed, non-probability, volunteer, web-based cross-sectional survey from April 3 to August 31, 2025. This pragmatic design was used because no comprehensive sampling frame was available and the study sought professionally and geographically diverse responses. Eligible participants self-reported a Korean medical license and at least 1 year of clinical experience. Recruitment used academic societies, professional associations, web postings, email distributions, and professional networks. Analyses included descriptive statistics, paired t-tests, chi-square tests, and modified Poisson regression with robust variance estimation.

**Results:**

Of 709 screening entries, 542 complete, eligible, non-duplicate questionnaires were analyzed. Respondents rated both physicians’ views (mean 4.68, SD 0.57) and patients’ views (mean 4.64, SD 0.59) highly in their own decision-making; physicians’ explanations received the highest rating among the predefined factors (mean 4.71, SD 0.60). Self-ratings of patient-centered communication and decision-making practices exceeded ratings of physicians in general (mean differences 0.22–0.27; all *p* < 0.001), but effect sizes were small (Cohen’s *d* = 0.25–0.29). Overall, 366 (67.5%) respondents reported having heard of SDM, 270 (49.8%) reported experience with SDM, and 211 (38.9%) rated their understanding as high or very high. Reported SDM term awareness was associated with self-reported experience (adjusted prevalence ratio 2.32, 95% CI 1.74–3.08; *p* < 0.001). Among 200 respondents who rated implementation as neutral or less feasible, 157 (78.5%) prioritized environmental factors.

**Discussion:**

Support for patient participation coexisted with substantial professional input, while reported familiarity and experience with SDM were not universal. Among respondents uncertain about implementation, structural conditions were the dominant perceived priority. Because volunteer sampling, self-report, and the cross-sectional design limit generalizability and causal inference, the policy implications are hypothesis-generating and require evaluation using representative, validated, observational, and longitudinal approaches.

## 1 Introduction

Shared decision-making (SDM) is a core component of patient-centered care and a health communication practice that supports informed patient participation (1). It is a collaborative process in which physicians and patients recognize that a decision is required, consider medically reasonable options, exchange information, and integrate the best available evidence with the patient’s values and preferences (2). SDM has been associated with improvements in patient knowledge, greater confidence, risk perception, and satisfaction with decisions (3) while promoting autonomy (4). Reflecting its growing importance, SDM has become a widely adopted model for clinical communication and decision-making and has been incorporated into clinical recommendations across specialties, encompassing rheumatology (5), cardiology (6), and oncology (7). Policy initiatives have also emphasized the importance and necessity of SDM (8). By supporting informed participation, SDM has broader public health relevance for health literacy, equity, patient safety, and the responsiveness of health services (8, 9).

Despite broad professional and policy support, routine implementation or broader adoption of SDM remains uneven. Barriers exist at multiple levels, including those related to patients, healthcare professionals, stakeholders (e.g., hospital management), institutions, or systems (10). Healthcare professional-related factors such as familiarity with SDM (11), communication skills (12), confidence in decision-support skills (13), and perceptions of patients’ willingness to participate (14) may influence implementation. However, these individual-level factors operate within clinical environments and may facilitate or constrain deliberative communication. Accordingly, time, clinical workflow, staffing, organizational support, and access to decision-support resources may be as important as healthcare professionals’ knowledge and attitudes (10, 15). These conditions are relevant to public health education and promotion because professional communication skills can be translated into routine practice only when service environments support meaningful patient participation (10, 15–17). Implementation research further suggests that SDM strategies cannot be fully understood independently of the healthcare context in which they are introduced. Consultation time, clinical workflow, staffing, reimbursement, and organizational support may shape whether SDM can be adopted in routine practice, and these contextual features influence the transferability of findings or strategies from one health system to another (16, 17).

Previous surveys have examined SDM across diverse national and clinical settings. A hospital-based survey in the Netherlands compared physicians’ preferred and usual decision-making roles and assessed their perceptions of the key components of SDM (18). A Japanese study examined perceived implementation factors among clinical research coordinators involved in clinical trials (19). A study conducted in a private healthcare network in Dubai assessed physicians’ self-reported SDM practices and physician-, patient-, relational, and environmental barriers (20). In the Philippines, a nationwide survey investigated the knowledge, attitudes, and practices of oncologists and oncology-related professionals regarding SDM (21). Several attempts have been made to examine healthcare professionals’ perceptions, and these studies differed in their participants, clinical settings, and measurement approaches, providing two key implications. First, additional multispecialty evidence is needed in South Korea on physicians’ views of clinical decision-making and related factors. Second, the findings from several countries underscore the importance of generating context-specific evidence rather than making direct cross-national prevalence comparisons.

Country-specific studies can contribute to international SDM literature by clarifying how implementation operates across different health systems and sociocultural contexts, rather than merely reporting local percentages. From a public health perspective, the key issue is whether routine health services create practical opportunities for patients to participate across settings, rather than only in isolated encounters. South Korea provides an informative setting because SDM must be integrated into a high-volume clinical environment in which brief consultations (22) and competing service demands may limit the time available for deliberative communication (23). Similar pressures occur to varying degrees across health systems, and consultation duration varies markedly between countries (24). Accordingly, evidence from South Korea may be relevant to broader questions about whether SDM awareness can translate into routine use when high patient volume, workflow constraints, and limited organizational capacity limit opportunities for patient participation. At the same time, the Korean context should not be treated as representing a single “Asian or Eastern model” of decision-making; preferences regarding the roles of patients, healthcare professionals, and family members vary within and between countries (25).

In South Korea, national physician surveys have described physicians’ experiences of medical encounters; however, SDM-specific evidence remains limited (2, 22). In particular, little is known about how physicians perceive the respective roles of patients and healthcare professionals in medical decision-making; how they evaluate their own communication and decision-making practices relative to those of physicians in general; whether they have heard of or used SDM; and which implementation domains they consider most important for improvement. This evidence gap limits the design of professional education and organizational strategies intended to strengthen patient participation at scale.

Accordingly, this study aimed to characterize South Korean physicians’ experiences and perceptions of clinical decision-making and SDM as a health communication practice; explore whether reported SDM term awareness and self-reported experience varied by physician and practice characteristics; and identify priorities for improving implementation. By examining these questions in a high-volume East Asian health system, this study seeks to provide context-specific evidence for public health education, professional practice improvement, and cross-national assessments of how organizational, professional, and health system conditions shape opportunities for patient participation.

## 2 Materials and methods

### 2.1 Study design

This study was a nationally distributed, non-probability, volunteer, web-based cross-sectional survey conducted in South Korea. It was designed to characterize physicians’ reported experiences and perceptions of clinical decision-making, with a particular focus on SDM as a health communication practice supporting patient participation in routine care.

Given the nationwide, multispecialty, and exploratory aims of this study, a self-administered web-based survey was used to efficiently capture physicians’ reported awareness, experiences, and perceptions across geographically dispersed settings. Self-reported outcomes were interpreted as physicians’ perceptions and experiences, rather than as direct measures of clinical behavior or objective knowledge.

### 2.2 Study population and eligibility

Eligible participants were physicians who self-reported holding a medical doctor’s license in South Korea and having at least 1 year of clinical experience. Eligibility was assessed using screening questions at the time of survey entry. Medical license numbers or external credentialing records were not used to verify eligibility; therefore, licensure status was based on self-reporting and could not be independently verified.

We sought to capture a wide range of perspectives across medical specialties and geographical regions in South Korea. Probability-based sampling was not feasible because the research team did not have access to a comprehensive national sampling frame with current contact information for eligible physicians. Therefore, we used non-probability volunteer recruitment through professional organizations and networks as a pragmatic measure to reach physicians across specialties, practice settings, and regions. This approach was chosen for exploratory characterization, increased heterogeneity, and hypothesis generation, and not for estimating nationally representative prevalence. An explicit diversification target of approximately 10 respondents per medical specialty was used to reduce concentration in only a few clinical fields. Geographic breadth and specialty targets were intended to diversify the volunteer sample; however, they did not establish representativeness. Because the primary objectives were descriptive and exploratory and no single confirmatory or causal hypothesis was prespecified, no formal *a priori* sample size or statistical power calculation was performed.

### 2.3 Questionnaire development and measures

The questionnaire was developed in three phases: literature review, qualitative consultation, and multistakeholder review. Development drew on previous surveys of healthcare professionals’ experiences and attitudes toward clinical decision-making (18–22) and Korean health service survey structures (26–28), which guided item content and organization. The initial draft was prepared by a multidisciplinary team comprising a family physician, two registered nurses, a medical sociologist, an ethicist, and a public health expert. Items were adapted from existing surveys, where appropriate. An independent expert panel comprising two physicians, a registered nurse, two medical students, and a public survey methodologist, none of whom participated in the initial drafting, assessed item relevance, clarity, comprehensiveness, and conceptual alignment. Items were revised by consensus. A quantitative content validity index was not calculated. The revised questionnaire was pilot-tested with 10 physicians to assess clarity and feasibility, and the pilot data were excluded from the final analysis. Typological errors were corrected, and wording was refined prior to finalization.

The final questionnaire comprised 58 items organized into four sections: (i) sociodemographic and professional characteristics (sex, age, clinical experience, specialty, practice setting, and position); (ii) experiences and perceptions of health communication during clinical decision-making; (iii) experiences and perceptions related to SDM; and (iv) additional items. The SDM section assessed whether respondents had heard of the term SDM, self-rated understanding, self-reported experience, perceived feasibility, and priority domains for improvement. Because the SDM definition was presented with the awareness item, this outcome was described as reported SDM term awareness rather than verified prior knowledge. Formal factor analysis and full construct validation of the questionnaire were not conducted. The instrument was designed as a multidomain survey with four sections organized as distinct factual and descriptive modules rather than a single latent scale. Accordingly, construct validity and broader psychometric performance of the questionnaire have not yet been established. Internal consistency was assessed only for four patient-centered decision-making behavior items—patient involvement, consideration of patients’ values and preferences, detailed information provision, and checking understanding—separately for the self-ratings and ratings of physicians in general. Cronbach’s alpha was used as a descriptive measure of item coherence (29). Satisfaction was excluded because it represented evaluation rather than behavior. SDM term awareness, understanding, and experience were treated as separate indicators rather than a single scale. English translations of the questionnaire items analyzed in this study are provided in Supplementary Data 1.

### 2.4 Recruitment and survey administration

Formal requests for cooperation in recruitment were sent to relevant academic societies and professional associations. Participating organizations disseminated the survey invitation by posting recruitment notices on their websites and emailing it to their members. No reminder messages were sent after the initial dissemination by the participating organizations. In addition, the research team circulated recruitment notices through professional networks. Interested individuals voluntarily accessed the eligibility screening page through a non-individualized web link included in this notice. An independent professional survey firm (30) hosted and administered the online survey platform. The survey was conducted from April 3 to August 31, 2025.

As recruitment notices were disseminated independently by the participating organizations and the research team, neither the total number of email invitations distributed nor the number of eligible physicians who viewed the website posts could be determined. Consequently, a conventional response rate based on an invitation or exposure denominator cannot be calculated. Therefore, we report on the disposition of survey entries from the initiation of eligibility screening to inclusion in the final analytical sample. Survey entry was defined as access that initiated the eligibility screening. Questionnaires were classified as complete only when the final survey page was submitted; entries discontinued before the final submission were classified as incomplete. Respondents who completed the questionnaire received mobile gift vouchers as compensation for their time.

### 2.5 Statistical analyses

All analyses were performed using SPSS version 27.0 and R version 4.4.3. Categorical variables were reported as frequencies and percentages, and continuous variables as means and standard deviations (SDs). For 5-point items, means, SDs, and 95% confidence intervals (CIs) were reported as descriptive summaries. Paired t-tests compared the respondents’ ratings of their own communication and decision-making practices with those of physicians in general. The association between self-reported SDM experience and combined reported SDM term awareness/self-rated understanding categories was evaluated using the Pearson chi-square test and Cramér’s V statistic. A modified Poisson regression with robust variance was used to estimate the adjusted prevalence ratios (aPRs) and 95% CIs for reported SDM term awareness and self-reported SDM experience (31, 32). The covariates selected *a priori* were sex, age per 10 years, practice setting, specialty group, and region. The SDM-experience model additionally included reported SDM term awareness. Primary analyses were unweighted. *Post hoc* raking weights were not used in the primary models because recruitment was non-probability-based and the available reference frame did not fully align with the analytic target population. Because the analyses were exploratory and involved multiple comparisons, *p*-values were interpreted as descriptive evidence rather than confirmatory hypothesis tests.

Practice settings were categorized into five pragmatic types: clinics or small hospitals, general hospitals, tertiary referral hospitals, nonclinical academic institutions, and other settings. Regarding the region, we classified 17 administrative districts into three pragmatic categories to reduce sparse cells: Seoul; other metropolitan areas, including Busan, Daegu, Incheon, Gwangju, Daejeon, Ulsan, and Sejong; and provinces comprising the remaining districts. All statistical tests were two-sided, and *p* < 0.05 was used as a descriptive threshold for the exploratory analyses.

### 2.6 Reporting guideline

This study was reported in accordance with the Strengthening the Reporting of Observational Studies in Epidemiology (STROBE) statement for cross-sectional studies (Supplementary Data 2) (33).

### 2.7 Ethical considerations

This study was approved by the Institutional Review Board (IRB) of Hanyang University, with which the authors were affiliated (IRB No. HYUIRB-202503-010). Before completing the survey, all participants were provided with detailed information about the study’s purpose and procedures via an online information sheet. Participants were informed that their participation was entirely voluntary and that they could withdraw from the survey at any time without submitting their responses.

Electronic informed consent was obtained before the eligibility screening. Respondents could proceed with the survey only after selecting the option that indicated their agreement to participate. They could discontinue participation at any time by closing the survey before the final submission; unsubmitted questionnaires were classified as incomplete. The requirement for written informed consent was waived by the IRB. Direct identifiers, including names and telephone numbers, were collected only from respondents who wished to receive mobile gift vouchers, by a professional survey firm via a separate form used solely for incentive delivery. These data were stored separately from the questionnaire responses, not included in the analytic dataset, and deleted within 30 days of incentive distribution. The research team had no access to direct identifiers.

## 3 Results

### 3.1 Participant flow

A total of 709 survey entries initiated the eligibility screening. Of these, 55 did not meet the eligibility criteria: 54 respondents reported not holding a medical license, and one had less than 1 year of clinical experience. Five submissions were flagged by the survey firm as duplicates, and 107 incomplete entries were excluded. The final analytical sample consisted of 542 complete, eligible, and non-duplicate questionnaires. Among the 649 eligible, non-duplicate survey starters, 542 submitted a complete questionnaire, corresponding to an 83.5% completion rate (Figure 1).

**Figure 1:**
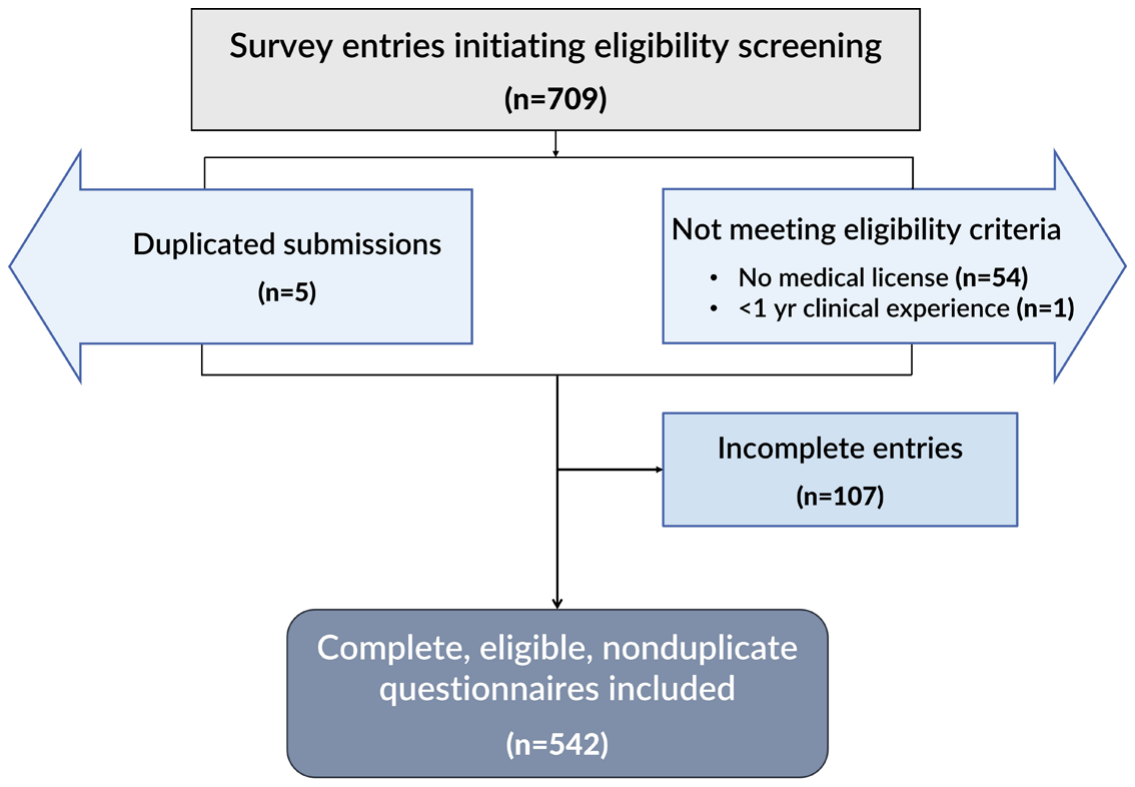
Flow of survey entries from initiation of eligibility screening to inclusion in the final analytic sample.

### 3.2 General characteristics

The final analytic sample included 542 respondents with a mean age of 40.6 years (SD 8.20) and mean clinical experience of 11.9 years (SD 8.78). Regarding practice settings, the largest proportion of participants worked in clinics or small hospitals and general hospitals (187/542, 34.5% each), followed by tertiary referral hospitals (132/542, 24.4%). Respondents were recruited from all 17 administrative districts, with 293 (54.1%) practicing in Seoul. In terms of position, 138 respondents (25.5%) were professors, and 125 (23.1%) were employed physicians without academic appointments. Internal medicine accounted for the largest proportion of participants (172/542, 31.7%), followed by surgical specialties (92/542, 17.0%), including general surgery, orthopedics, neurosurgery, thoracic and cardiovascular surgery, and plastic surgery. The detailed characteristics are shown in Table 1 and Figure 2.

**Table 1:** General characteristics of participants (*N* = 542).

Category and variable	Participants, *n* (%)
Sex	
Male	305 (56.3)
Female	237 (43.7)
Age (years)	
30–39	265 (48.9)
40–49	181 (33.4)
50–59	70 (12.9)
20–29	14 (2.6)
≥ 60	12 (2.2)
Clinical experience (years)	
5–9	206 (38.0)
< 5	96 (17.7)
10–14	92 (17.0)
≥ 25	68 (12.5)
20–24	42 (7.7)
15–19	38 (7.0)
Practice setting	
Clinics or small hospitals	187 (34.5)
General hospital	187 (34.5)
Tertiary referral hospital	132 (24.4)
Non-clinical academic institution	26 (4.8)
Others	10 (1.8)
Position	
Professor (faculty)	138 (25.5)
Employed physician (non-faculty)	125 (23.1)
Private practitioner	118 (21.8)
Resident or intern	89 (16.4)
Fellow	59 (10.9)
Others	13 (2.4)
Region	
Seoul	293 (54.1)
Province	151 (27.9)
Other metropolitan areas	98 (18.1)

**Figure 2:**
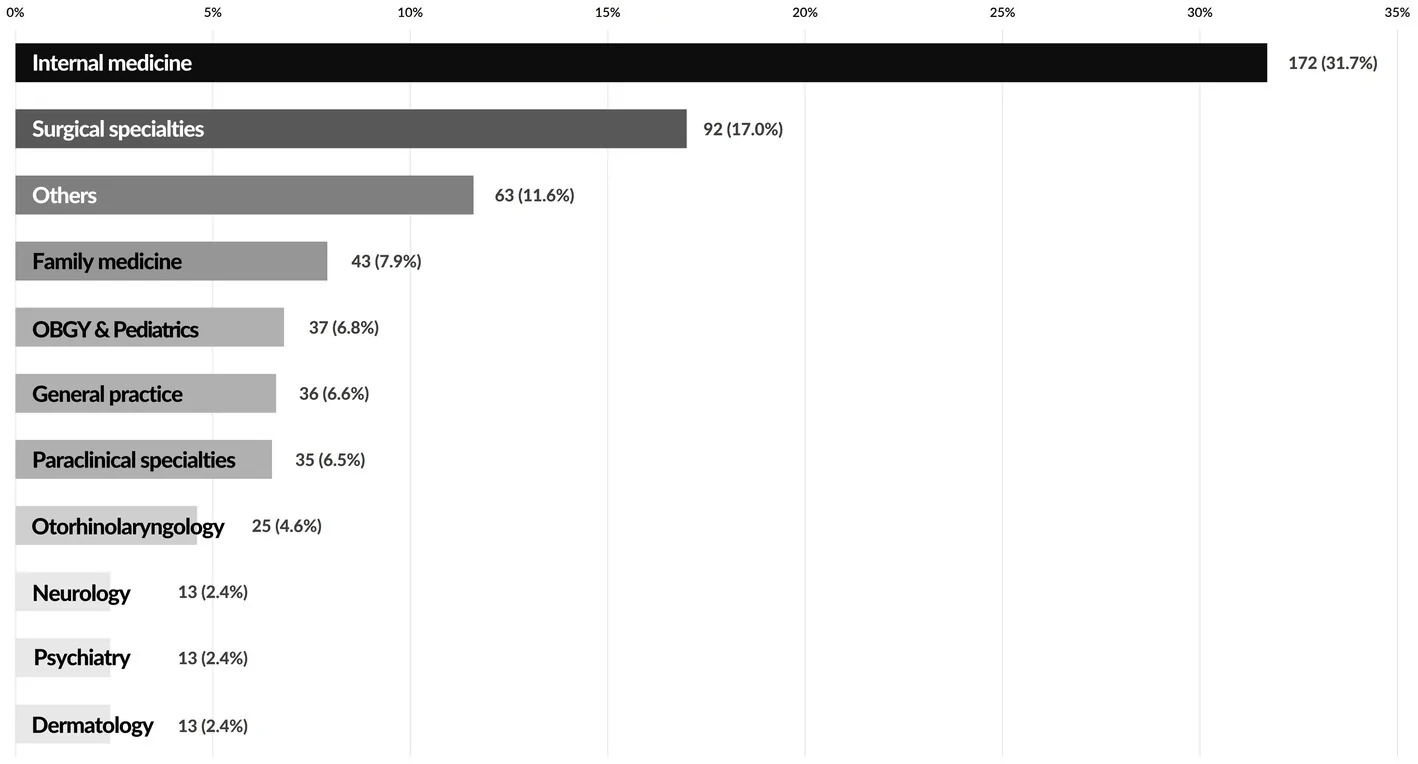
Distribution of participants by medical specialty. Bars show the percentage of the analytic sample (*N* = 542). Paraclinical specialties include radiology, pathology, laboratory medicine, nuclear medicine, occupational and environmental medicine, and preventive medicine. OBGY, obstetrics and gynecology.

### 3.3 Perceived importance of primary stakeholders’ perspectives and decision-making factors

When asked which stakeholder perspectives they believed were important to patients during clinical decision-making, physicians gave the highest mean rating to patients’ views (mean 4.75, SD 0.56), followed by physicians’ own views and judgments (mean 4.67, SD 0.56). Regarding physicians’ own clinical decision-making, their views and judgments (mean 4.68, SD 0.57) and patients’ views (mean 4.64, SD 0.59) were both rated highly. Compared with the importance-to-patients frame, the mean rating assigned to patients’ views was 0.11 points lower under the own-practice frame (95% CI 0.06–0.16; *p* < 0.001), whereas the rating assigned to physicians’ own views did not differ significantly between the two frames (mean difference −0.02, 95% CI − 0.07 to 0.03; *p* = 0.478) (Table 2).

**Table 2:** Perceived importance of primary stakeholders’ perspectives: to patients vs. to physicians’ clinical decision-making (*N* = 542).

Primary Stakeholders	Importance to patients, mean (SD)	Importance to their own clinical decision-making, mean (SD)	Mean difference (95% CI)	Cohen’s *d*	*p*
Patient	4.75 (0.56)	4.64 (0.59)	0.11 (0.06–0.16)	0.18	<0.001
Physician	4.67 (0.56)	4.68 (0.57)	−0.02 (−0.07–0.03)	−0.03	0.478
Patient’s family	4.09 (0.75)	4.02 (0.77)	0.07 (0.01–0.13)	0.09	0.028
Other healthcare professionals	3.50 (0.96)	3.40 (1.02)	0.10 (0.01–0.17)	0.13	0.002

SD, standard deviation; CI, confidence interval. Differences were calculated as ratings of importance to patients minus ratings of importance in respondents’ own clinical decision-making.

Among the factors considered important in the respondents’ clinical practice, explanations provided by physicians received the highest rating (mean 4.71, SD 0.60), followed by patient values and preferences (mean 4.48, SD 0.71). Information obtained from television and internet sources received the lowest rating (mean 2.69, SD 1.29) (Figure 3).

**Figure 3:**
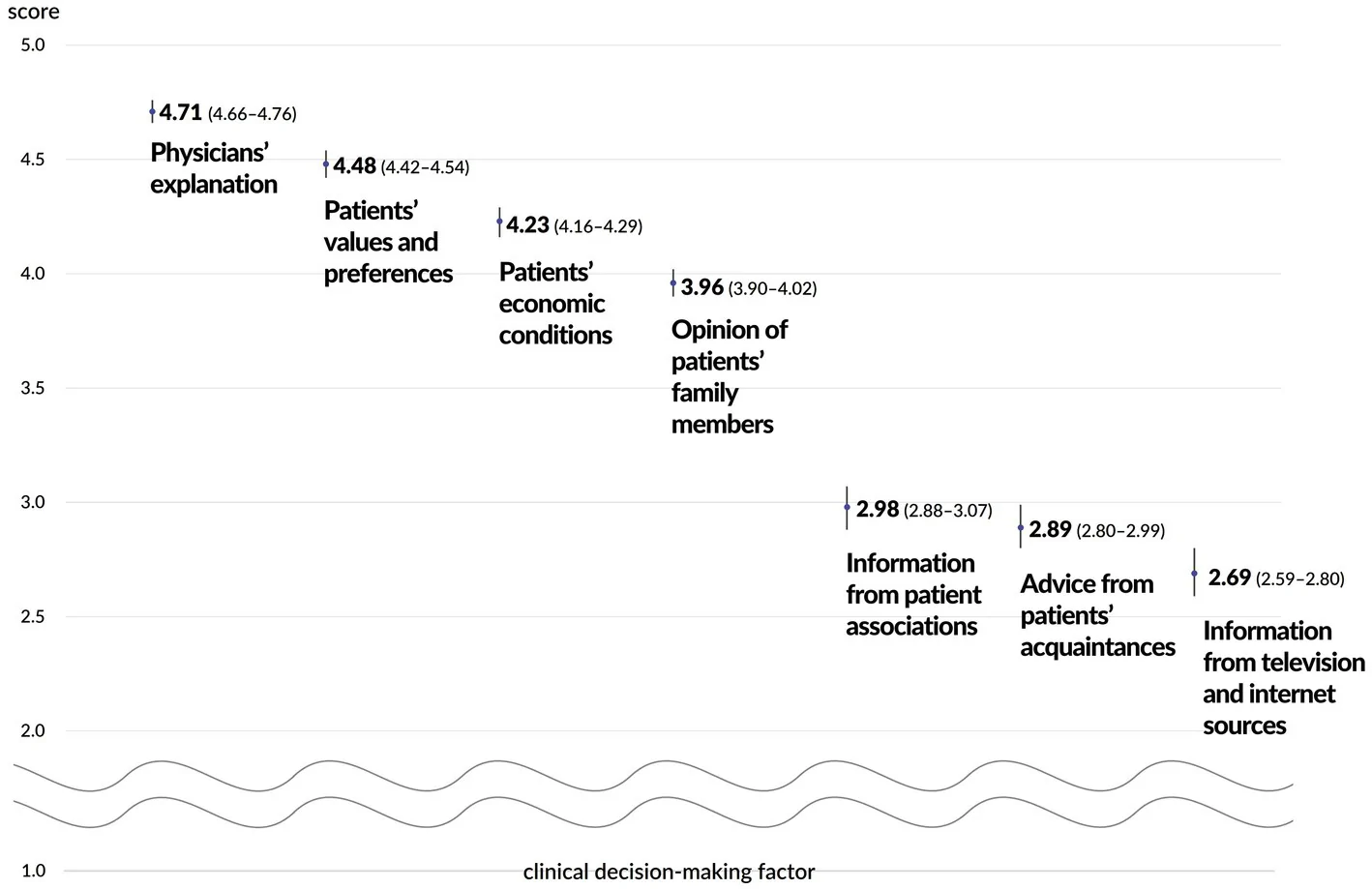
Physician-rated importance of clinical decision-making factors in their own clinical practice. Values are presented as means with 95% confidence intervals.

### 3.4 Patient-centered decision-making behaviors: self vs. physicians in general

Table 3 shows the differences between self-ratings and ratings of physicians in general regarding patient-centered decision-making behaviors. Physicians rated their own communication and decision-making practices more favorably than those of physicians in general across all four behavioral items. All differences were statistically significant (*p* < 0.001). Mean paired differences ranged from 0.22 to 0.27, with small effect sizes (Cohen’s *d* = 0.25–0.29). The four-item composite differed by 0.94 (95% CI 0.72–1.16; Cohen’s *d* = 0.36). Cronbach’s alpha was 0.723 for self-ratings and 0.856 for ratings of physicians in general. These findings represent comparative self-reports rather than observed clinical performance.

**Table 3:** Comparison of self-ratings and ratings of physicians in general for patient-centered decision-making bahaviors (*N* = 542).

Statements	Self, mean (SD)	Physicians in general, mean (SD)	Mean difference (95% CI)	Cohen’s *d*	*p*
Involving patients in clinical decision-making when appropriate	4.17 (0.64)	3.95 (0.83)	0.22 (0.15–0.29)	0.26	<0.001
Considering patients’ values and preferences when making clinical decisions	4.15 (0.70)	3.91 (0.86)	0.23 (0.16–0.31)	0.26	<0.001
Providing patients with detailed information about their medical condition and options	4.05 (0.71)	3.83 (0.89)	0.22 (0.14–0.29)	0.25	<0.001
Checking patients’ understanding of the provided information	4.04 (0.75)	3.77 (1.01)	0.27 (0.19–0.35)	0.29	<0.001

Internal consistency for four items was calculated. Cronbach’s α = 0.723 for self-ratings and Cronbach’s α = 0.856 for ratings of physicians in general. Differences were calculated as self-ratings minus ratings of physicians in general.

### 3.5 Experience, awareness, and understanding regarding SDM

Table 4 presents participants’ self-reported experience by reported term awareness and self-rated understanding. Overall, 67.5% (366/542) of respondents reported having heard of the term SDM, and 49.8% (270/542) reported experience with SDM. Among those who reported term awareness, 57.7% (211/366) rated their understanding as high or very high; this corresponded to 38.9% (211/542) of the total sample. Self-reported SDM experience was strongly associated with the combined term-awareness/understanding categories (χ^2^_3_ = 130.0, *p* < 0.001; Cramér’s V = 0.492). Among respondents who reported experience with SDM, 61.1% (165/270) rated their understanding as high, compared with 16.9% (46/272) among those who reported no experience with SDM. Because the SDM definition accompanied the term-awareness item, the 41 respondents who reported experience despite no reported term awareness may have recognized similar practices without knowing the label; these responses should not automatically be treated as inconsistent.

**Table 4:** Self-reported SDM experience by reported SDM term awareness and self-rated understanding (*N* = 542).

Experience with SDM	No reported term awareness (*n* = 176)	Reported term awareness, low understanding (*n* = 36)	Reported term awareness, moderate understanding of SDM (*n* = 119)	Reported term awareness, high understanding (*n* = 211)	Total (*N* = 542)
Yes (*n* = 270)	41 (15.2)	7 (2.6)	57 (21.1)	165 (61.1)	270
No (*n* = 272)	135 (49.6)	29 (10.7)	62 (22.8)	46 (16.9)	272
Total (*N* = 542)	176	36	119	211	542

SDM, shared decision-making. Values are presented as n (row %), except for the total row and total column. Pearson χ^2^_3_ = 130.0, p < 0.001; Cramér’s V = 0.492.

In the adjusted model shown in Table 5, females had a higher prevalence of reported SDM term awareness than males (aPR 1.53, 95% CI 1.35–1.73; *p* < 0.001). Reported term awareness was also more prevalent among respondents in tertiary referral hospitals than among those in clinics or small hospitals (aPR 1.23, 95% CI 1.06–1.42; *p* = 0.006) and among respondents in provinces than among those in Seoul (aPR 1.15, 95% CI 1.01–1.30; *p* = 0.038). Respondents in diagnostic/support/population-based specialties had a lower prevalence of reported term awareness than those in medical/generalist specialties (aPR 0.67, 95% CI 0.51–0.88; *p* = 0.004). Self-reported SDM experience was more prevalent among females (aPR 1.35, 95% CI 1.13–1.62; *p* < 0.001), respondents in general hospitals (aPR 1.31, 95% CI 1.07–1.62; *p* = 0.009), and respondents in tertiary referral hospitals (aPR 1.34, 95% CI 1.08–1.66; *p* = 0.008) than in their respective reference groups. Reported SDM term awareness was strongly associated with self-reported experience (aPR 2.32, 95% CI 1.74–3.08; *p* < 0.001). However, these associations should be considered exploratory and should not be interpreted causally.

**Table 5:** Adjusted associations with reported SDM term awareness and self-reported SDM experience (*N* = 542).

Predictor	Reported term awareness, aPR (95% CI)	*p*	Self-reported SDM experience, aPR (95% CI)	*p*
Female (vs male)	1.53 (1.35–1.73)	<0.001	1.35 (1.13–1.62)	<0.001
Age (per 10 years)	1.08 (0.99–1.17)	0.075	1.10 (0.99–1.22)	0.066
General hospital (vs clinic/small hospital)	1.11 (0.97–1.27)	0.132	1.31 (1.07–1.62)	0.009
Tertiary referral hospital (vs clinic/small hospital)	1.23 (1.06–1.42)	0.006	1.34 (1.08–1.66)	0.008
Nonclinical academic institution (vs clinic/small hospital)	0.93 (0.62–1.39)	0.723	1.07 (0.63–1.81)	0.816
Other settings (vs clinic/small hospital)	1.02 (0.57–1.83)	0.939	1.08 (0.50–2.29)	0.850
Surgical/procedural (vs medical/generalist)	1.05 (0.92–1.19)	0.498	0.95 (0.79–1.14)	0.581
Diagnostic/support/population (vs medical/generalist)	0.67 (0.51–0.88)	0.004	0.76 (0.53–1.09)	0.142
Other metropolitan areas (vs Seoul)	1.14 (0.98–1.33)	0.079	1.05 (0.85–1.30)	0.660
Province (vs Seoul)	1.15 (1.01–1.30)	0.038	0.97 (0.81–1.18)	0.790
Reported SDM term awareness: yes (vs no)			2.32 (1.74–3.08)	<0.001

SDM, shared decision-making; aPR, adjusted prevalence ratio; CI, confidence interval. Modified Poisson regressions with robust variance were conducted. Primary analyses were unweighted because the volunteer sample was nonprobability-based and the available reference frame was not aligned with the analytic target population. The model for self-reported SDM experience was additionally adjusted for reported term awareness. Medical/generalist specialties comprise general practice, internal medicine, neurology, psychiatry, pediatrics, dermatology, family medicine, and emergency medicine; surgical/procedural specialties comprise all surgical specialties, obstetrics and gynecology, ophthalmology, otorhinolaryngology, and urology; and diagnostic/support/population specialties comprise the remainder.

### 3.6 Perceived barriers to implementing SDM

Among the 542 participants, 200 (36.9%) rated the feasibility of implementing or expanding SDM as neutral or lower. Environmental factors were most frequently selected as the highest-priority domain for improvement (157/200, 78.5%), followed by healthcare professional-related factors (32/200, 16.0%). Within the environmental domain, short consultation or discussion times and insufficient reimbursement for explanation and counseling were each selected by 83 respondents (41.5%), followed by a busy or inefficient clinical environment (62/200, 31.0%). These findings describe perceived priorities within this conditional subgroup rather than objectively measured determinants of SDM.

## 4 Discussion

### 4.1 Principal findings

This nationally distributed, non-probability, volunteer, web-based survey identified three patterns of public health communication and patient participation. First, respondents assigned high importance to patient input in clinical decision-making, while also placing importance on physicians’ views and explanations. Second, the reported SDM term awareness and self-reported experience were not universal and varied across physician and practice characteristics. Third, among the respondents who rated the feasibility of implementing or expanding SDM as neutral or lower, environmental or system-level factors were most frequently selected as the highest-priority domains for improvement. Respondents also rated their own communication and decision-making practices slightly more favorably than those of physicians in general, but the effect sizes were small. Within this sample, these patterns framed SDM as both a clinical skill and a professional practice and service-environment issue.

Stakeholder ratings should be interpreted as reflecting perceived importance rather than as evidence of actual decision-making behavior. The findings suggest that physicians viewed clinical decision-making as involving both patient and professional input, but they did not establish how decisions were actually made, how much influence patients had, or whether the final authority remained physician-centered. From a public health communication perspective, meaningful participation requires not only information provision but also opportunities for patients to ask questions, compare options, and express their values (2). Direct observation, patient-reported measures, consultation recordings, and analysis of actual decision-making partners are required to examine the distribution of decision-making authority in medical encounters.

SDM findings are best understood as a reported term-awareness–experience gradient rather than a definitively established knowledge–practice gap. Reported awareness of the term SDM was more common than self-reported experience, and comprehension was assessed by self-rating rather than by an objective knowledge test. Because the definition of SDM accompanied the awareness item, this measure did not establish objective prior knowledge. Therefore, the findings indicate that familiarity, confidence in understanding, and self-reported experience of SDM were not universal in this heterogeneous physician sample. Direct numerical comparisons with previous international studies may not be appropriate because the surveys involved different populations, clinical settings, and measurement strategies. A study in the Netherlands focused on hospital physicians’ preferred and usual roles (18); a study in Japan involved clinical research coordinators (19); a study in Dubai was conducted within a private hospital network (20); and a study in the Philippines focused on oncology-related professionals (21). Collectively, however, these studies support the broader point that the conceptual endorsement of SDM does not necessarily translate into routine implementation.

Prioritization of environmental factors is particularly relevant to implementation. This result is consistent with international evidence that SDM uptake is shaped not only by physicians’ knowledge and skills, but also by clinical workflow, consultation time, staffing, organizational resources, professional roles, and organizational conditions (10). From a public health perspective, these service conditions may shape whether opportunities for patient participation are routinely available across institutions and populations. The present study did not objectively test whether consultation duration, reimbursement, workload, or decision-support infrastructure led to lower SDM experience. Instead, these factors should be viewed as candidate implementation mechanisms that warrant further evaluation through prospective comparative studies.

Differences in self–other ratings should also be interpreted with caution. Respondents rated their own practices more favorably than those of physicians in general, a pattern compatible with a better-than-average effect in comparative self-evaluation (34). However, the differences were small and based on self-reporting rather than observed behavior. Therefore, these findings should not be taken as proof of inaccurate self-assessment, resistance to feedback, or cognitive barriers to SDM implementation. A more conservative interpretation is that physicians’ self-perceptions and their perceptions of physicians in general may not be fully aligned and that future studies should compare self-ratings with patient-reported or observer-rated measures of communication.

### 4.2 International relevance and transferability

The international and public health relevance of the findings lies less in the absolute percentages observed in this Korean volunteer sample than in the suggested implementation patterns. The prominence of systemic priorities aligns with the view that SDM uptake depends on interactions between interventions, implementation strategies, organizational settings, and the wider health system context (10, 17). This convergence raises a broader question for other countries: can health care professional education alone produce routine SDM when consultation time, workload, workflow, reimbursement arrangements, and the decision-support framework do not support deliberative communication? South Korea therefore provides an informative case for other high-volume health care systems facing comparable implementation pressures.

However, the Korean findings should not be assumed to apply directly to all settings. The relative roles assigned to patients, primary caregivers, and physicians and differences across specialties and practice settings may depend on local professional norms, financing structures, care pathways, and individual preferences (35). Cross-national research has shown that family involvement preferences vary within countries and across broadly defined cultural regions (25). Therefore, the present results should be used to generate comparative questions rather than universal conclusions. Researchers and policymakers in other countries should compare the Korean source context with their own populations, healthcare service delivery structures, and institutional conditions when judging the relevance of these findings or adapting SDM strategies (16, 17).

### 4.3 Practical implications

From a public health education and promotion perspective, these findings underscore the need for multilevel implementation strategies that critically evaluate contextual barriers and facilitators, rather than claiming effectiveness of any single intervention. Prior evidence highlights the importance of physician education, adequate consultation time, streamlined clinical processes, team-based support, and incentives that foster deliberative communication (36). Yet, the present cross-sectional self-reports cannot establish whether modifying these conditions would directly enhance SDM. Consequently, educational, organizational, or financing approaches should be tailored to local contexts and tested prospectively, rather than advanced as definitive policy recommendations based solely on this survey.

Training programs can combine conceptual instruction with simulation (37), structured feedback (38), patient-reported measures (39), consultation recordings (40), or observer-based assessment (41). These approaches may help physicians to compare their perceptions with more direct evidence of communication practices. However, such feedback should be framed as professional development and quality improvement rather than as evidence of cognitive bias, which was not established in this study. Training modules have been developed as components of broader SDM adoption strategies, but their effect may depend on the organizational context in which they are delivered (17, 42).

The most transferable implication is that healthcare systems should assess whether their clinical environments enable SDM. Countries with high patient volumes or short consultation times may find the Korean experience especially relevant, whereas systems with different financing, staffing, and consultation structures may require different combinations of educational, organizational, and policy interventions. Future cross-national studies should use harmonized definitions and measures to determine whether the reported term-awareness–experience gradient, prioritization of environmental factors, and variation across practice settings recur under different health system conditions. Because opportunities for participation may differ across institutions and patient groups, public health research should also examine whether structural constraints contribute to inequities in access to SDM.

### 4.4 Strengths and limitations

This study has several strengths. Recruitment reached respondents in all 17 administrative districts in South Korea and included a broad range of specialties, positions, and practice settings. This geographic and professional breadth broadened the range of perspectives captured but did not make the volunteer sample fully nationally representative. This study examined multiple related but distinct aspects of clinical decision-making and SDM, including stakeholder importance, self–other ratings, reported SDM term awareness, self-rated understanding, self-reported experience, implementation feasibility, and priority improvement domains. Reporting eligibility screenings, incomplete questionnaires, and duplicate exclusions improved the transparency of the web-based survey. Integrating individual, interpersonal, and organizational dimensions supported the public health interpretation of the findings.

This study has some limitations. First, recruitment used a non-probability volunteer approach through academic societies, professional associations, website posts, email distributions, and professional networks. As neither the total number of invitations distributed nor the number of eligible physicians exposed to recruitment notices was known, a conventional response rate could not be calculated (43) and nonresponse bias could not be quantified. Selection bias may have affected sample composition. Physicians with a greater interest in communication, patient-centered care, or SDM may have been more likely to participate. The findings should therefore not be interpreted as nationally representative prevalence estimates or generalized to all South Korean physicians without further probability-based or otherwise well-characterized sampling (44, 45). Coverage across all 17 administrative districts is breadth only and does not establish national representativeness.

Second, eligibility was based on self-reported medical licensure and clinical experience, and could not be independently verified. All outcomes were reported by physicians at a single time point, making the results susceptible to recall, social desirability (46), and common method bias (47). Self-reported SDM experiences do not verify whether SDM occurs or establish its frequency or quality. Self-rated understanding should not be interpreted as objective SDM knowledge, and ratings of physicians in general do not reflect actual observed peer performance.

Third, the questionnaire underwent qualitative expert review and pilot testing, but no formal factor analysis or construct validation of the full 58-item instrument was conducted. Although internal consistency was calculated for the four patient-centered behavior items, Cronbach’s alpha alone did not establish construct validity (48). Therefore, measurement errors and construct misclassifications were possible. Several variables, including specialties, practice settings, and regions, were collapsed into pragmatic analytical categories to avoid sparse cells, which may have obscured meaningful within-group variations. Regression analyses were exploratory, and the cross-sectional design could not establish temporal order or causal relationships. Accordingly, public health and policy implications are hypothesis-generating rather than evidence that specific educational, organizational, or financing changes will necessarily improve SDM.

Finally, barrier analysis was restricted to respondents who rated implementation feasibility as neutral or lower, and the study included only physicians. Therefore, the study could not determine whether physicians’ perceptions corresponded to patients’ experiences or population-level differences in participation opportunities. Future studies should include perspectives of other healthcare professionals and patients’ family members, use validated instruments to assess SDM knowledge and behavior, compare self-reports with patient-reported and observer-rated consultation measures, and employ longitudinal or interventional designs to assess temporal relationships and intervention effects.

## 5 Conclusion

In this non-probability volunteer sample, physicians placed high value on patient participation, but familiarity with SDM terminology and self-reported experience were not universal. Among respondents uncertain about implementation feasibility, environmental constraints were most often prioritized. These findings cannot be interpreted as national prevalence estimates or as evidence that structural conditions caused differences in reported SDM experience. They identified physician education, consultation time, clinical workflow, reimbursement, and organizational support as potential targets for prospective evaluation. Future probability-based or otherwise well-characterized surveys and longitudinal or interventional studies should incorporate other perspectives, validated instruments, and observed SDM behaviors to assess its implementation and transferability across healthcare settings.

## Data Availability

The original contributions presented in the study are included in the article/Supplementary material; further inquiries can be directed to the corresponding author.
