# Supplementary material for: Physicians’ perceptions, reported experience, and perceived structural barriers to shared decision-making in South Korea: a multispecialty survey
Source: Front Public Health. 2026 Oct 15;14:1938324. doi: 10.3389/fpubh.2026.1938324 (PMC13716757; doi:10.3389/fpubh.2026.1938324)
Supplement: Supplementary file 1 [file Supplementary_file_1.DOCX]

Supplementary Data 1. English translation of the questionnaire items analyzed in the study

*This file provides an English translation of the questionnaire items analyzed in this study.*

*The full questionnaire, containing additional items not used in the present analyses,
is therefore not reproduced in full here.*

# 1 Perceived importance to patients in clinical decision-making

*The following questions ask about who or which factors are important to patients during clinical decision-making. Please select the most appropriate response.*

*Scale: 1 = Not at all important; 2 = Not important;
3 = Neither important nor unimportant; 4 = Important; 5 = Very important*

## 1.1 Please indicate how important you think the views and judgments of the following key stakeholders are to patients in clinical decision-making.

| **Primary stakeholders** | **Importance** |
| --- | --- |
| The patient |  |
| The patient’s family |  |
| The physician |  |
| Other healthcare professionals (eg, nurses) |  |
| Other, please specify: ______________ |  |

## 1.2 Please indicate how important you think the following factors are to patients in clinical decision-making.

| **Factors** | **Importance** |
| --- | --- |
| The patient’s values, preferences, and beliefs |  |
| The patient’s family’s opinion |  |
| Advice of the patient’s acquaintances |  |
| Explanation of physicians |  |
| Information from patient associations |  |
| Economic conditions |  |
| Other, please specify: ______________ |  |

# 2 Communication practices during clinical consultations

*The following statements concern communication practices during clinical consultations. Please indicate the extent to which you agree or disagree with each statement.*

*Scale: 1 = Strongly disagree; 2 = Disagree;
3 = Neither agree nor disagree; 4 = Agree; 5 = Strongly agree*

## 2.1 Please answer each statement based on your own experience.

| **Statements** | **Agreement** |
| --- | --- |
| During clinical consultations, I provide patients with detailed information about their medical condition and options. |  |
| During clinical consultations, I check whether patients understand the information provided. |  |
| For making clinical decisions, I consider patients’ values and preferences. |  |
| When appropriate, I involve patients in clinical decision-making. |  |
| During clinical consultations, I am satisfied with information provision and decision-making. |  |

## 2.2 Please answer each statement based on your perception of physicians in general, excluding yourself.

| **Statements** | **Agreement** |
| --- | --- |
| During clinical consultations, physicians provide patients with detailed information about their medical condition and options. |  |
| During clinical consultations, physicians check whether patients understand the information provided. |  |
| For making clinical decisions, physicians consider patients’ values and preferences. |  |
| When appropriate, physicians involve patients in clinical decision-making. |  |
| During clinical consultations, physicians are satisfied with information provision and decision-making. |  |

# 3 Awareness, experiences, and perceived barriers to shared decision-making

## 3.1 Have you ever heard of shared decision-making?

*Shared decision-making in healthcare is a collaborative communication process among healthcare professionals, patients, and their primary caregivers to make decisions that reflect patients’ preferences and values by comparing multiple medically valid options based on evidence and clinical judgment.*

1) Yes

2) No

## 3.2 [Only for respondents who selected 1) in Q3.1] How would you rate your overall understanding of shared decision-making?

1) Very low

2) Low

3) Moderate

4) High

5) Very high

## 3.3 Have you ever used shared decision-making during clinical consultations?

1) Yes

2) No

## 3.4 In your view, how feasible is the current implementation or broader adoption of shared decision-making in clinical practice in South Korea?

1) Not feasible at all

2) Not feasible

3) Neutral

4) Feasible

5) Highly feasible

## 3.5 [Only for respondents who selected 1)–3) in Q3.4.] If the implementation or broader adoption of shared decision-making is difficult or uncertain, which of the following categories of factors should be prioritized for improvement?

1) Patient-related factors

2) Physician-related factors

3) Environment-related factors

4) Other, please specify: ______________

## 3.6 [Only for respondents who selected 1)–3) in Q3.4.] Which patient-related factors should be prioritized for improvement? Please select two.

1) Limited patient willingness to participate in decision-making

2) Patient reliance on family members or physicians for decision-making

3) Patient beliefs or values that conflict with medical evidence

4) Inappropriate family involvement

5) Patient distrust resulting from information overload

6) Other, please specify: ______________

## 3.7 [Only for respondents who selected 1)–3) in Q3.4.] Which physician-related factors should be prioritized for improvement? Please select two.

1) Insufficient provision of information to patients

2) Passive physician attitudes toward patient involvement

3) Insufficient consideration of patients’ family preferences and opinions

4) Lack of communication skills training for physicians

5) Avoidance of responsibility for providing explanations

6) Use of medical jargon in patient communication

7) Other, please specify: ______________

## 3.8 [Only for respondents who selected 1)–3) in Q3.4.] Which environment-related factors should be prioritized for improvement? Please select two.

1) Short consultation time

2) Inefficient clinical workflow and organizational constraints

3) Insufficient consideration of patients’ family preferences and opinions

4) Healthcare payment system

5) Inadequate financial reimbursement for patient counseling and explanation

6) Lack of decision-support tools or systems

7) Heavy clinical workload and physician fatigue

8) Other, please specify: ______________

# 4 Background and professional characteristics

## 4.1 Do you hold a valid medical doctor’s license in South Korea?

1) Yes

2) No

## 4.2 Please indicate your total duration of clinical practice experience to date.

______ years ______	months

## 4.3 What is your sex?

1) Male

2) Female

3) Other (please specify: ______________)

## 4.4 What is your date of birth?

Please write your date of birth.

(Month) ______ (Day) ______	(Year) ______

## 4.5 Which region do you work in?

1) Seoul

2) Busan

3) Daegu

4) Incheon

5) Gwangju

6) Daejeon

7) Ulsan

8) Sejong

9) Gyeonggi-do

10) Gangwon-do

11) Chungcheongbuk-do (North Chungcheong)

12) Chungcheongnam-do (South Chungcheong)

13) Jeollabuk-do (North Jeolla)

14) Jeollanam-do (South Jeolla)

15) Gyeongsangbuk-do (North Gyeongsang)

16) Gyeongsangnam-do (South Gyeongsang)

17) Jeju-do

## 4.6 Which of the following best describes your current primary workplace or practice setting?

1) Clinic

2) Hospital with 30 or more beds

3) General hospital with 100 or more beds

4) Tertiary general hospital

5) Long-term care hospital

6) Public health institution (eg, public health center)

7) Medical college (including graduate school of public health)

8) No affiliated institution

9) Other, please specify: ______________

## 4.7 Please specify your medical specialty.

*General practitioners without board certification should enter “General Practitioner.”*

___________________

## 4.8 Which of the following best describes your current professional position?

1) Private practitioner

2) Employed physician (non-faculty)

3) Professor (faculty physician)

4) Fellow or subspecialty trainee

5) Resident or intern

6) Public health doctor

7) Military medical officer

8) Not currently engaged in clinical practice

9) Other (please specify: ______________)
